# Sol–gel derived ceramic nanoparticles as an alternative material for microstrip patch antenna in WLAN applications

**DOI:** 10.1038/s41598-024-63454-5

**Published:** 2024-06-13

**Authors:** Sekhar Didde, R. S. Dubey

**Affiliations:** 1 Department of Electronics & Communication Engineering, Swarnandhra College of Engineering and Technology, Seetharamapuram, Narsapur, A.P. India; 2 SR University, Ananthasagar, Hasanparthy, Hanumakonda District, Warangal, T.S. 506372 India

**Keywords:** Return loss, Voltage standing wave ratio, Microstrip patch antenna, Electrical and electronic engineering, Materials for devices, Nanoscale materials

## Abstract

In the fast-evolving realm of communication technology, microstrip patch antennas (MPAs) are in high demand owing to their compact size, lightweight, inexpensive, ease of integration, and compatibility with modern electronic devices. This research focuses on the synthesis of ZnAl_2_O_4_Ca (ZAC) ceramic nanoparticles using an economical sol–gel method suitable for microstrip patch antenna applications. The structural analysis study of ZAC nanoparticles confirmed the polycrystalline nature with 8.1 nm of crystallite size whereas an investigation of functional groups showed the corresponding vibration modes. Morphological investigation revealed the spherical grains having their mean diameter of 12.32 nm. The dielectric property’s examination, revealed the dielectric permittivity of 13, loss tangent of 0.02, and conductivity of 67 μΩ^−1^ cm^−1^. Furthermore, a prototype patch antenna fabricated using ZAC ceramics demonstrated a dual-band performance at frequencies 2.8 GHz and 4.8 GHz, with return losses of − 25.78 dB and − 28.5 dB, respectively. This work suggests the suitability of ZAC ceramic nanoparticles for use in WLAN applications.

## Introduction

Due to increasing demand and rapid advancements in real-time communication systems, multiband antennas with wide bandwidth are becoming increasingly favorable due to their ability to provide high radiation patterns. The primary objective of multiband antennas is to ensure minimal interference with other frequency bands. In the ever-expanding landscape of wireless communication technology, certain key attributes are highly required. These include low-profile, lightweight construction, and the improvement of band designations such as the S-band from 2 to 4 GHz, C-band from 4 to 8 GHz for, Wireless LAN (WLAN) from 2.4 to 5 GHz, WiFi operating at 2.4 GHz, and X-band covering the 8–12 GHz range^1^. The crucial parameters for describing the microwave characteristics of a communication system include a high-quality factor (Qxf) helpful to wideband designations, an appropriate permittivity (ε_r_) to facilitate miniaturization, and a temperature coefficient of resonant frequency (τ_f_) to ensure the stability and reliability of microwave devices. Microwave communication technology has impacted cellular mobile communication, television broadcasting, radar technology, WiFi modules, signal tracking systems, cavity resonators, filters, isolators, circulators, and signal jamming systems. The primary factor behind this progress can be attributed to the utilization of microwave dielectric ceramics^2^.

Microwave-absorbing materials are gaining significant attention for their dual role in mitigating electromagnetic pollution in civilian settings and enhancing stealth capabilities in military applications. Researchers explored synthesis and applications of various nanomaterials such as CuCr_2_O_4_, La-substituted CuFe_2_O_4_, CuFe_2_O_4_, and ZnAl_2_O_4_, to enhance microwave absorption and reflection loss^3–6^. Additionally, studies on capped BaFe_2_O_4_ nanoparticles and expanded-carbon microspheres blended with ZnAl_2_O_4_ nanoparticles demonstrated an excellent absorption property, suggesting their potential for practical applications^7,8^. Presently, magnetic ferrites are highly valued for their properties such as high resistivity, moderate permeability, and low electric and magnetic losses, making them crucial across various applications. In this context, researchers highlighted the potential of Cu-substituted Ni–Zn nanocrystalline ferrites for industrial and domestic applications, while another work on CoSm ferrite-metamaterial absorbers evidenced promising electromagnetic features with potential applications in satellite communication^9,10^. Furthermore, a study on Gd–Ce-substituted garnet ferrites, Al-doped spinel and garnet nanoferrites showed potential for industrial and technological applications in different frequency bands^11,12^.

ZnAl_2_O_4_ nanoparticles possess exceptional electrical, mechanical, and optical properties, making them ideal for microwave applications. Researchers explored the applications of doped ZnAl_2_O_4_ nanoparticles for the enhanced thermal stability, mechanical strength, band gap, electrical properties and so on. Furthermore, synthesis approach such as the sol–gel method is a low-temperature and straightforward one. This technique yields nanoparticles with their uniform size distribution and allows ease incorporation of small-sized dopants^13,14^. Numerous researchers have undertaken the synthesis, characterization, and investigation of ceramic nanoparticles. For instance, Yin et al. employed the solid-state route method to synthesize Ba_2_CuGe_2_O_7_ ceramic nanoparticles doped with Mg. They investigated the microwave dielectric properties of Ba_2_CuMgGe_2_O_7_ sintered at 900 °C, and reported, ε_r_ = 9.43 and τ_f_ = -76 ppm/°C along with Qxf = 20,000 GHz^15^. Wu et al. synthesized and characterized, Li_2_WO_4_ and composite Ba_3_Ti_4_Nb_4_O_2_ microwave dielectric ceramic nanoparticles using the solid-state reaction method. The ceramic material exhibited, ε_r_ = 21.85 and τ_f_ = − 7.78 ppm/°C along with Qxf = 10,534 GHz^16^. Jiamao et al. adopted the stearic acid method to synthesize NdAlO_3_ ceramic nanoparticles at low temperature. They obtained ε_r_ = 23.02, Qxf = 65,320 GHz, and τ_f_ = − 32.4 ppm/°C^17^. Similarly, Mengjuan et al. prepared (1 − x)CoTiNb_2_O_8_-xZnNb_2_O_6_ ceramic material via the conventional solid-state reaction route to mitigate τ_f_ value. This material revealed, ε_r_ = 39.2, Qxf = 40,013 GHz, τ_f_ = 3.57 ppm/°C^18^. In another study, Jiamao et al. employed the stearic acid route method to synthesize SmAlO_3_ and examined its dielectric properties. By sintering at 1500 °C for 4 h, they achieved a consistent microstructure with an average grain size of 1.8 μm. The measured values of dielectric parameters were ε_r_ = 20.22, Qxf = 74,110 GHz, and τ_f_ = − 74.6 ppm/°C^19^. Likewise, Hsiang et al. explored the preparation of low-temperature co-fired Ca_0.7_Nd_0.2_TiO_3_ ceramic material incorporating CaO-B_2_O_3_-SiO_2_. Their investigation revealed ε_r_ and Qxf values of 22.8 and 2380 GHz, respectively^20^. Further, Rahman et al. employed the sol–gel method to synthesize Co_x_Zn_(0.90−x)_Al_0.10_Fe_2_O_4_ ceramics for MPA applications. They observed the variation in crystallite size, dielectric permittivity, and tangential loss from 24.45 to 20.84 nm, 3.5 to 4.5, and 0.002 to 0.006, respectively with an increased Co concentration from 20 to 60%. The antenna of this composite exhibited a return loss (RL) varying from − 35 to − 26 dB, with a bandwidth of 2.20–2.5 GHz, at a resonant frequency (F_r_) between 4.6 and 4.0 GHz^21^. Borah et al. employed magnetodielectric composites doped with cobalt ferrite for MPA applications whose average grain size was 10 nm. The real part of the dielectric permittivity and permeability were found to vary from 1 to 2.905 and 1.01 to 1.05, respectively, with an increased concentration from 1% volume fraction to 5% volume fraction. Additionally, they fabricated MPA, which exhibited an RL of − 19.45 dB at the resonance frequency of 9.5 GHz^22^. In a similar fashin, Ashish et al. prepared a miniaturized MPA using barium hexaferrite (BaFe_12_O_19_) through the co-precipitation method. They reported ε_r_ = 6.2 and ε_o_ = 1.9, with an average crystallite size of 60 nm. They subsequently constructed MPA with BaFe_12_O_19_, which showed its RL of − 25 dB at F_r_ of 4.25 GHz with a bandwidth of 166 MHz^23^. In other study, the same group reported the synthesis of Ni_0.5_Zn_0.3_Co_0.2_In_0.1_Fe_1.9_O_4_ nanoparticles for miniaturized microstrip antenna application and also investigated dielectric permittivity and permeability values of 5.3 and 5.5, respectively. The fabricated antenna demonstrated a remarkable RL of − 42 dB at the resonance frequency of 450 MHz^24^. Bhongale at el. fabricated Mg–Nd–Cd ferrite-based MPA prepared by the co-precipitation method having a grain size of 1.37 µm. The dielectric permittivity, permeability, and tangential loss were measured to be 3.18, 1.29, and 0.2, respectively. The fabricated MPA exhibited a return loss of − 19.16 dB at a frequency of 9.35 GHz, with a voltage standing wave ratio (VSWR) of 1.26^25^.

Among above-discussed materials, doped ZnAl_2_O_4_ is promising for increasing its dielectric permittivity, which facilitates the miniaturization of patch antennas, resulting in compact antenna designs suitable for integration into various electronic devices, such as smartphones, tablets, and wearable technology. Additionally, it reduces the dielectric loss indicating less signal energy lost in the form of heat within the substrate. This mechanism is fruitful in enhancing the antenna’s efficiency and minimizing signal distortion. Furthermore, Ca doping can be beneficial for tuning the thermal expansion coefficient of ZnAl_2_O_4_ so that thermal stresses which causes performance degradation can also be minimized. In this manner, doped zinc aluminate, as substrate material, exhibits excellent thermal, electrical, and chemical stability. This makes antennas fabricated with it suitable for harsh operating environments, including outdoor and industrial settings.

In this study, we synthesize Zn_0.988_Al_2_O_4_Ca_0.012_ ceramic nanoparticles using the sol–gel method and present a comprehensive study of various characteristics, including structural, morphological, and dielectric properties. Additionally, we fabricated a prototype MPA, which exhibited remarkable return loss values in both the S-band and C-band frequencies, measured to be − 25.78 dB and − 28.5 dB, respectively. Consequently, our research highlights the suitability of the prepared ceramic nanoparticles for dual-band MPA applications, unambiguously for the S-band and C-band. Notably, there is no prior similar work reported in the literature, particularly in the context of the same material and its application for dual-band communication, like WLAN. “Materials and Methods” Section presents the materials and methods employed in this study. A comprehensive study of results is discussed in “Results and Discussion” Section. Lastly, “Conclusions” Section reviews the findings.

## Materials and methods

For the synthesis of ZnAl_2_O_4_Ca ceramic nanoparticles, various chemicals including C_2_H_5_OH (Ethanol, Sigma-Aldrich), Al(NO_3_)_3_.9H_2_O (Aluminum Nitrate Nonahydrate, purity of 99.99%, Sigma-Aldrich, C_2_H_6_O_2_ (Ethylene Glycol, SdFine), Zn(O_2_CCH_3_)_2_.2H_2_O (Zinc Acetate Dihydrate, purity of 99.99%, Lobychem), Ca(NO_3_)_2_H_8_O_4_ (Calcium Nitrate Tetrahydrate, purity of 99.99%, Sigma-Aldrich, and HNO_3_ (Nitric Acid, Lobychem) were used as purchased.

**Figure 1:**
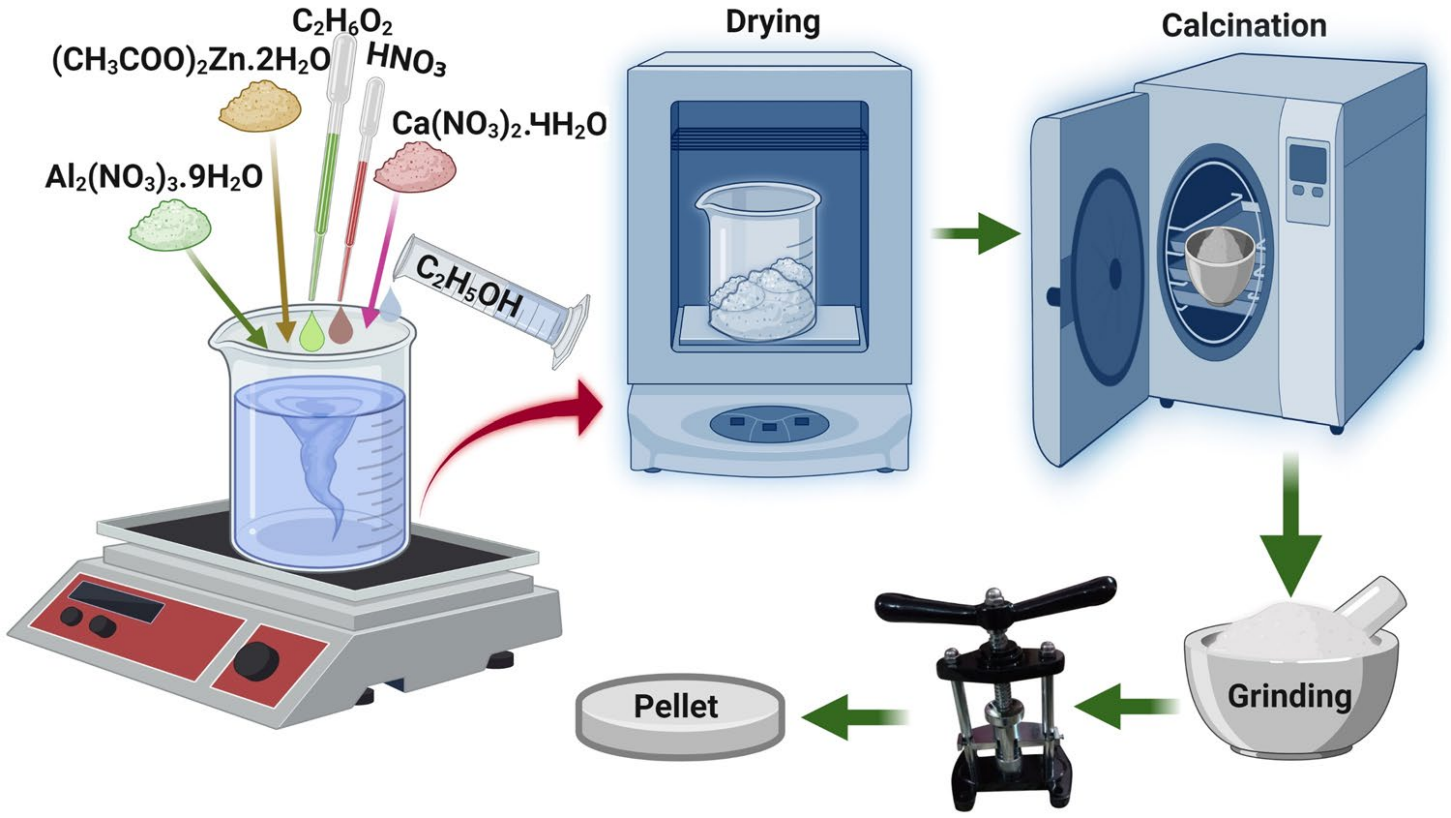
The process of preparation of ZAC ceramic nanoparticles. Reprinted with permission of Springer Nature © 2022^26^.

To prepare Ca doped zinc aluminate (Zn_(1−x)_Al_2_O_4_-Ca_x_) ceramic nanoparticles with molar concentration x = 0.012, the following synthesis procedure was employed, and the prepared sample named as ‘ZAC’ throughout the manuscript. The synthesis procedure is illustrated in Fig. 1. Initially, 60 ml ethanol combined with 22.5 gm aluminum nitrate nonahydrate was stirred to get dissolved solution. Subsequently, 0.49 ml ethylene glycol and 10.87 gm zinc acetate were introduced into the solution. After stirring for 5 min, we added 0.17 gm calcium nitrate tetrahydrate under stirring at 75 °C for 1 h. Following this, we introduced 0.36 ml nitric acid into the solution and later kept it for drying at 180 °C for 1 h. The dried powder was further processed by calcining it in a furnace for 2 h at 900 °C and then the powder was finely ground to get fine ZAC nanoparticles.

The prepared nanoparticles were examined using various techniques: X-ray Diffractometer (XRD, Bruker D8, Venture, Germany) for probing the crystallographic structure, Fourier-Transform Infrared Spectroscopy (FTIR, Perkin Elmer Spectrum Two, US) for analyzing the chemical composition, Transmission Electron Microscopy (TEM, Talos F200S G2, US) coupled with Energy-dispersive X-ray spectroscopy (EDS) for studying the morphology and compositional elements in the sample, LCR Meter (PSM 1735 N4L, Newtons 4th Limited, UK) for evaluating the electrical properties of the ZAC pellet (having diameter of 10 mm and thickness of 1.5 mm), and Vector Network Analyzer (VNA, R&S ZVL, Germany) for measuring the scattering parameters of the microstrip patch antenna.

## Results and discussion

**Figure 2:**
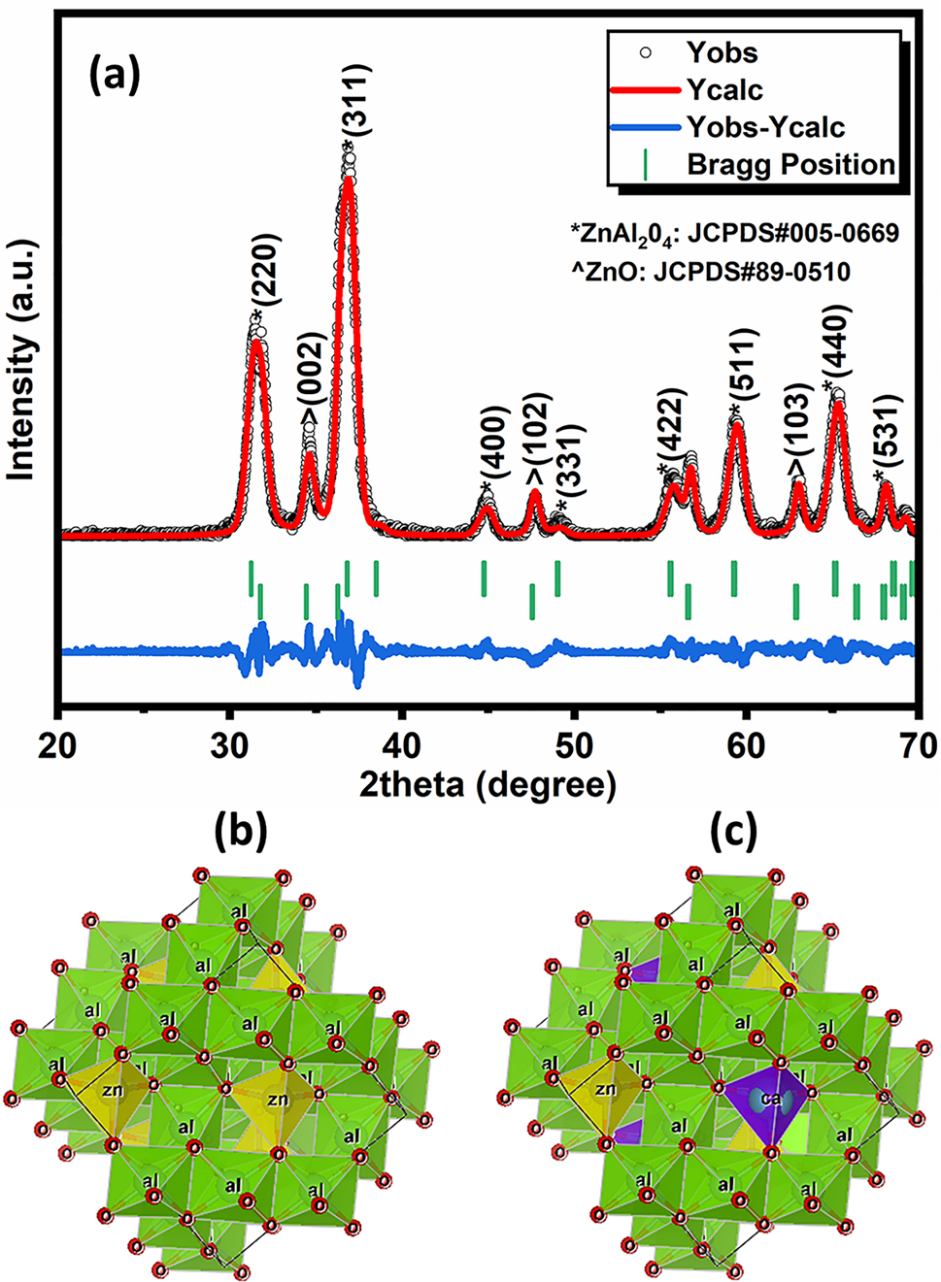
Rietveld refinement plot of the XRD pattern of ZAC ceramic nanoparticles (**a**), representation of crystal structure of undoped (**b**) and Ca-doped ZnAl2O4 (**c**).

Figure 2a depicts the Rietveld refinement plot of the XRD pattern of ZAC ceramic nanoparticles, which revealed its polycrystalline characteristics. The intensity peaks, indexed at 2θ values of 31.49°, 36.8°, 44.9°, 47.7°, 49.0°, 55.68°, 56.8°, and 59.28° were attributed to the crystal planes of (220), (311), (400), (331), (331), (422), (422), and (511), respectively, which correspond to the characteristics of ZnAl_2_O_4_. Additionally, another peak observed at 2θ = 34.65° was attributed to the ZnO plane of (101).

These characteristic peaks in the XRD pattern closely matched those found in #JCPDS Card Nos. 005-0669 and 89-0510. The crystallite size was calculated to be 8.1 nm using Scherer’s equation. Figure 2b,c illustrate the visualization of crystal structures of undoped and Ca-doped ZnAl_2_O_4_ obtained using the Vesta package.

The crystal structure of undoped ZnAl_2_O_4_ shown in Fig. 2b shows the presence of individual elements such as Zn, Al and O, while an additional element, Ca (dopant) is evident in the crystal structure of Ca–ZnAl_2_O_4_ as depicted in Fig. 2c. Table 1 summarizes the crystal structure parameters obtained from the Rietveld refinement analysis. It revealed the unit cell parameters for gahnite as a = b = c = 8.07098 Å, and for zincite, a = b = 3.24170 ≠ c = 5.18760 Å. The cell volume for gahnite was noticed to be 525.7494 (Å)^3^, and 47.2110 (Å)^3^ for zincite.

**Table 1:** Structural parameters obtained from the Rietveld refinement of XRD pattern.

Crystal system	Cubic	Hexagonal
Space group	F d -3 m	P 63 m c
Space Number	227	186
Phase	Gahnite	Zincite
Unit cell (Å)		
a	8.07098	3.24170
b	8.07098	3.24170
c	8.07098	5.18760
Volume (Å)^3^	525.7494	47.2110
Angle (degree)		
α	90.000	90.000
β	90.000	90.000
γ	90.000	120.000

**Figure 3:**
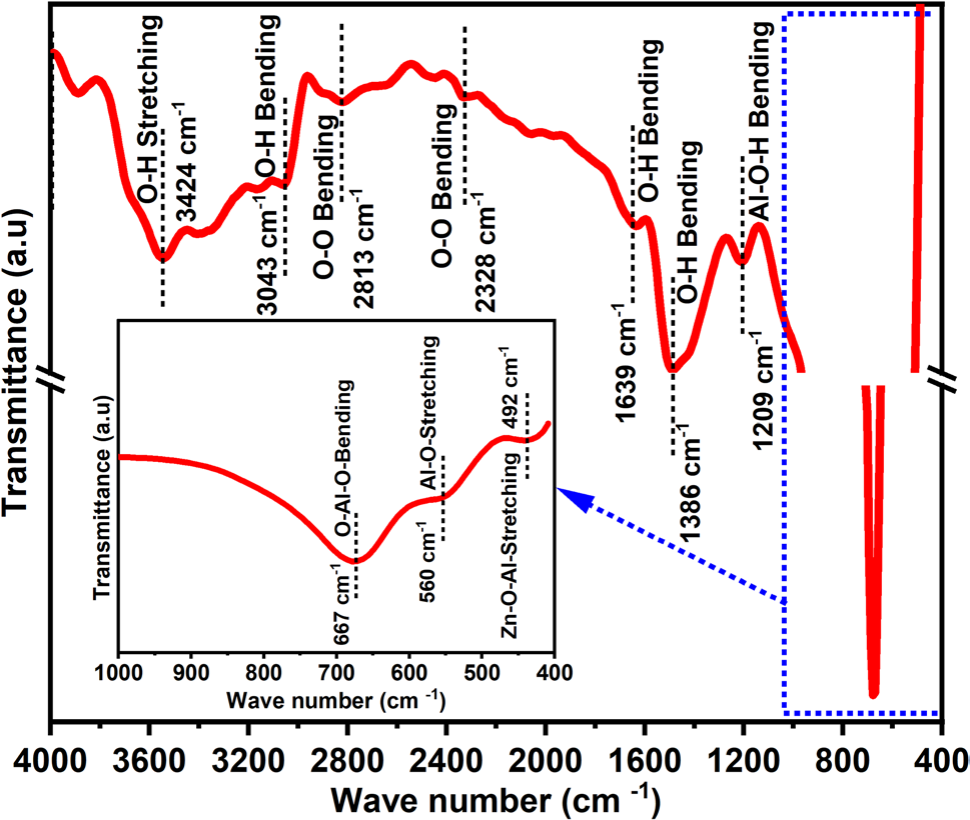
FTIR spectrum of ZAC ceramic nanoparticles.

Figure 3 depicts the FTIR spectrum of Ca-doped ZnAl_2_O_4_ ceramic nanoparticles in the wave number range of 400–4000 cm^−1^. Several vibrational peaks were evident at various wavenumbers, specifically at 492, 560, 667, 1209, 1386, 1639, 2328, 2813, 3043, and 3424 cm^−1^, corresponding to different functional groups. The peaks at 3424, 3043, 1639, and 1386 cm^−1^ were found associated with the stretching vibration modes of O–H, contributed by the water molecules. Another stretching peaks, resulting from the O–O bond observed at 2813 and 2328 cm^−1^ were found associated with the FCC crystal lattice of oxygen atoms. Another peak noticed at 1209 cm^−1^ was assigned to Al–O–H indicating stretching vibration mode. In the low-frequency region below 1000 cm^−1^, peaks originating at 8667, 560, and 492 cm^−1^ were found associated with the mineral network. This complex region can be attributed to the stretching frequencies of aluminum-oxygen (Al-O) and metal–oxygen-aluminum (M–O–Al) bonds. Specifically, the band 492 cm^−1^ corresponds to the spinel characteristics of ZnAl_2_O_4_ and originated due to the Zn–O–Al, Al–O, and Zn–O modes^27–31^.

**Figure 4:**
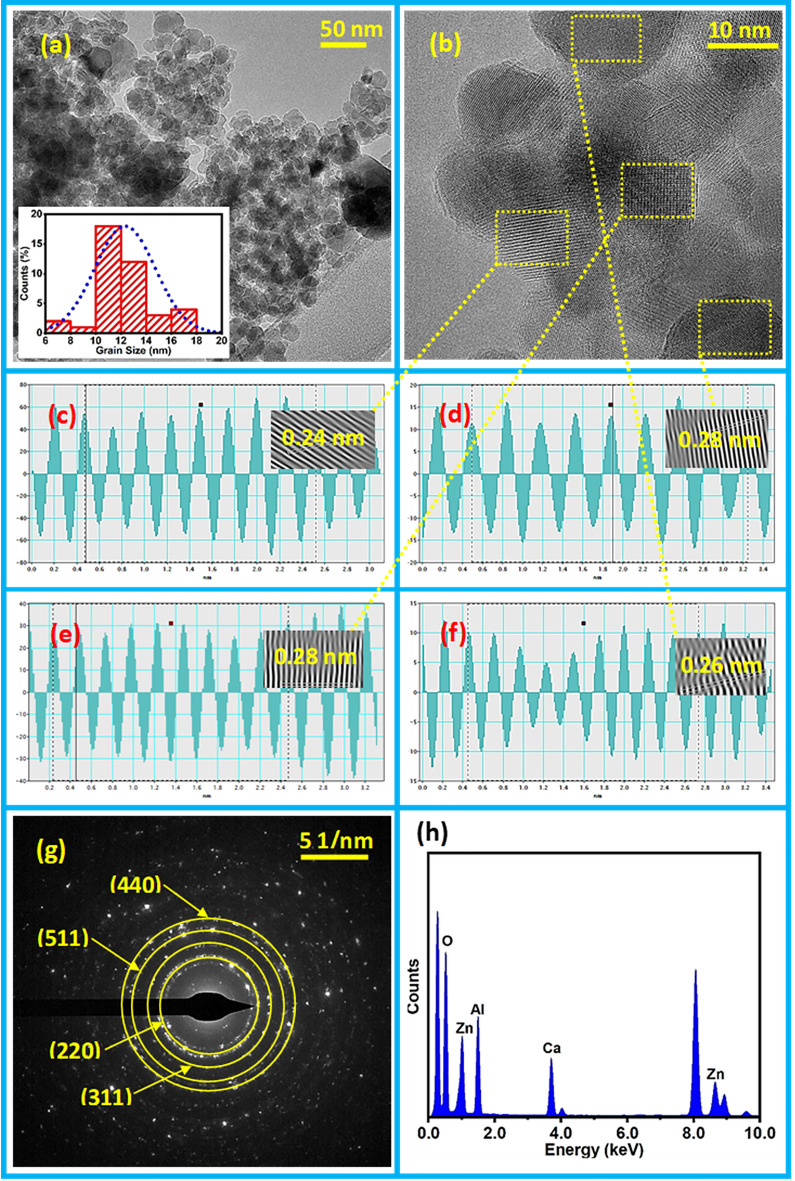
TEM micrograph with a histogram (**a**), HRTEM image (**b**) line-profile with an inset inverse FFT (**c**–**f**), SAED pattern (**g**) and EDS spectrum (**h**).

The morphology of ZAC nanoparticles was examined using TEM. Figure 4 illustrates TEM and HRTEM micrographs along with an inverse FFT lattice and line-profile analyses, SAED pattern, and EDS spectrum. In Fig. 4a, a TEM micrograph of the prepared sample revealed spherical grains arranged with their clear boundaries. An inset histogram shows the distribution of grains. The estimated mean diameter of the grains was 12.32 nm. In Fig. 4b, a high-resolution TEM image provides a detailed view of the d-spacing planes with marked rectangular boxes. We performed the inverse FFT process of the lattices and conducted line-profiling, as depicted in Fig. 4c–f. The obtained d-spacing values were 0.24 nm and 0.28 nm, corresponding to ZnAl_2_O_4_ planes (311) and (220) along with 0.26 nm of ZnO plane (002). Figure 4g shows the selected area electron diffraction (SAED) pattern, confirming the co-centric solid rings that represent the polycrystalline nature of the ZAC nanoparticles. This SAED pattern aligns well with the XRD pattern depicted in Fig. 2a. The elemental composition of ZAC analyzed by EDS is depicted in Fig. 4h. This spectrum reveals elemental peaks corresponding to Zn, Al, O, and Ca, identified at energy levels of 8.6 keV, 1.49 keV, 0.5 keV, and 4.1 keV, respectively.

The comparison of obtained structural and morphological parameters with previously reported literature is presented in Table 2.

**Table 2:** Comparison of parameters of different ceramic materials.

Synthesis approach	Materials	Crystallite size (nm)	Particles/grains size (nm)	Calcination temperature (°C)	References
Co-precipitation	MgxCd_1−x_Fe_2_O_4_	40.59	2	1500	^32^
Hydrothermal	Zn_1−x_Mn_x_Al_2_O_4_	–	13	200	^33^
Sol–gel	NiAl_2_O_4_	12	13–15	450	^34^
Hydrothermal	Zn_0.95−x_Cr_0.05_Mn_x_Al_2_O_4_	14.20	23	700	^35^
Hydrothermal	Zn_0.95_Cr_0.05_Al_2_O_4_	21	25	700	^36^
Sol–gel	ZnAl_2_O_4_Ca	8.1	12.32	900	Present Work

**Figure 5:**
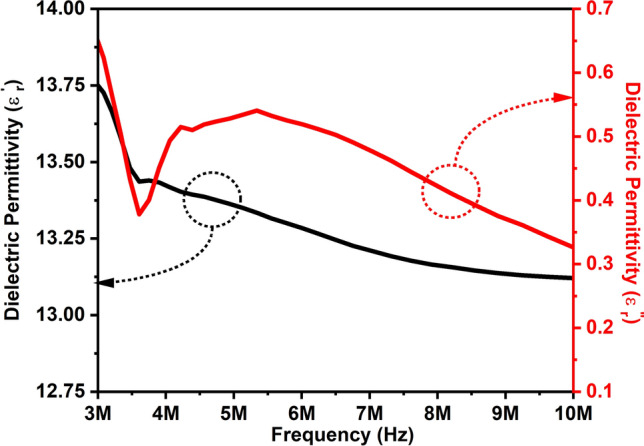
Dielectric permittivity (real and imaginary) of ZAC ceramic nanoparticles.

The study of real and imaginary values of dielectric permittivity is significant in analyzing the behavior of ZAC compound. The real part of it signifies energy storage capability, whereas the imaginary part indicates energy dissipation by the material. We have performed both analyses as plotted in Fig. 5 which illustrates the real and imaginary dielectric permittivity of ZAC over a frequency range from 3 to 10 MHz.

The dielectric permittivity was estimated using ε_r_’ = Ct/(ε_o_S), where ε_r_’ represents the relative dielectric permittivity, C is the capacitance, t is its thickness, ε_o_ is the free space dielectric permittivity, and S is the surface area of the sample. From this figure, it is evident that the dielectric permittivity decreases as the frequency rises. This behavior aligns with the principles of the Maxwell–Wagner model and Koop's phenomenological theory. Specifically, the real dielectric permittivity was observed to be 13.75 at 3 MHz and 13 at 10 MHz. The imaginary dielectric permittivity decreases as the frequency increases. This phenomenon is attributed to polarization resonance. At lower frequencies, polarization resonance requires more energy, leading to higher energy absorption. Conversely, at higher frequencies, resonance occurs more easily with lower polarization, resulting in less energy absorption. This behavior aligns with the principle underlying the observed changes in the imaginary dielectric permittivity.

The imaginary dielectric permittivity of the ZAC ceramic nanoparticles was determined to be 0.62 and 0.32 at frequency 3 and 10 MHz, respectively using the formula ε_r_’’ = ε_r_’ tanδ, where ε_r_’ represents the real dielectric permittivity and tanδ represents the dielectric loss. For the study of dielectric properties, the selection of this frequency range was based on the frequency limitation of the LCR meter. However, the goal of this investigation was to observe the typical dielectric behavior of ZAC nanoparticles.

**Figure 6:**
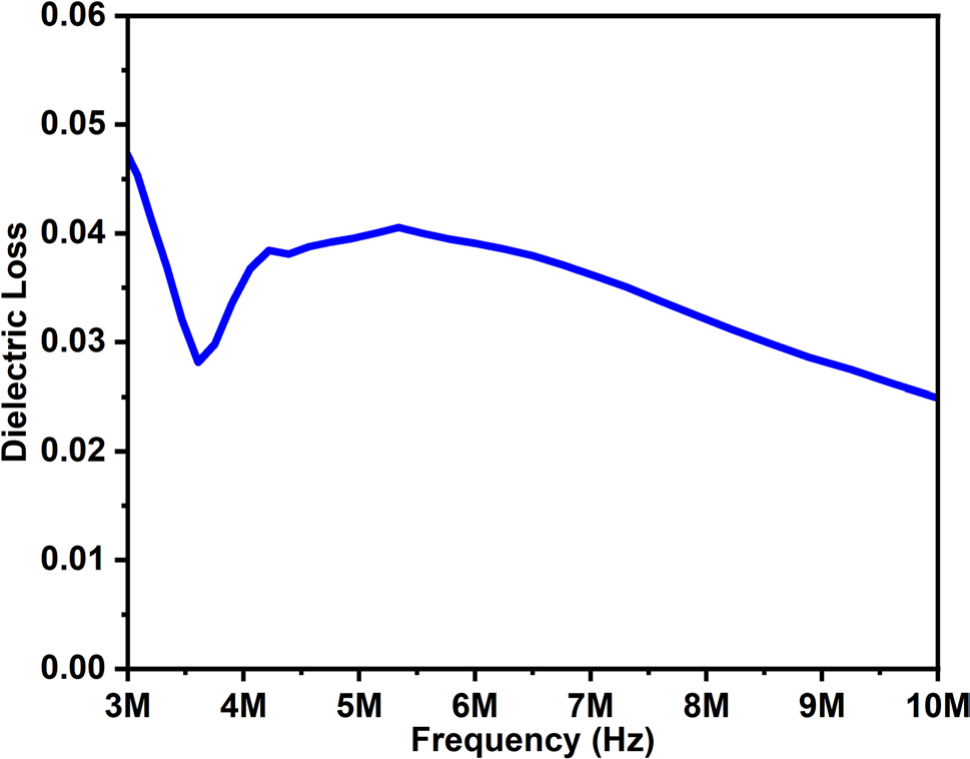
The dielectric loss of ZAC ceramic nanoparticles.

Dielectric loss (tanδ), arises when the electric field remains constant with the polarization of dielectric materials. Figure 6 illustrates the dielectric loss of the ZAC sample. Due to domain wall resonance, the dielectric loss decreases at higher frequencies. The tangent loss values were 0.045 and 0.024, corresponding to the frequency values 3 and 10 MHz.

**Figure 7:**
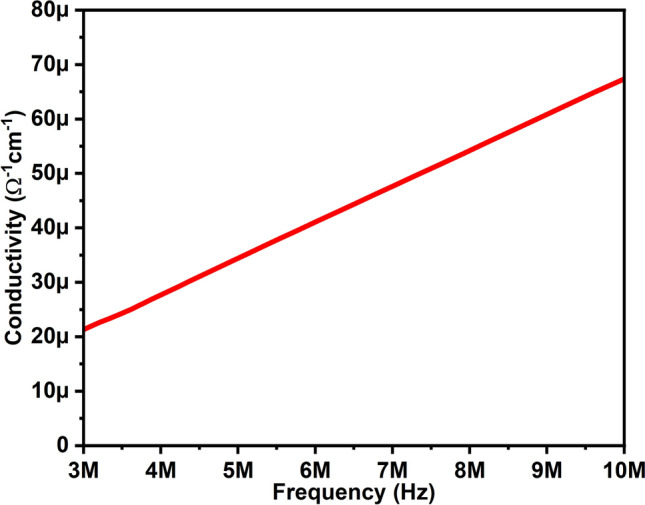
Conductivity of ZAC ceramic nanoparticles.

Figure 7 illustrates the conductivity of the ceramic nanoparticles over a frequency range from 3 to 10 MHz. Conductivity was determined using σ = ε_r_ε_o_ωtanδ, where ω is the angular frequency, ε_r_ is the relative dielectric constant, ε_o_ is the dielectric constant for free space, and tanδ is the dielectric loss. An observed trend in Fig. 7 shows that conductivity increases with higher frequencies. This behavior can be attributed to the migration of electrons. Grain boundaries act as potential barriers opposing the movement of charged particles, in line with the Maxwell–Wagner interface model. At higher frequencies, electrons have sufficient energy to overcome the barrier potential, leading to higher conductivity^37,38^. The calculated conductivity values were 21.84 and 67.33 µm at frequencies 3 and 10 MHz, respectively.

**Figure 8:**
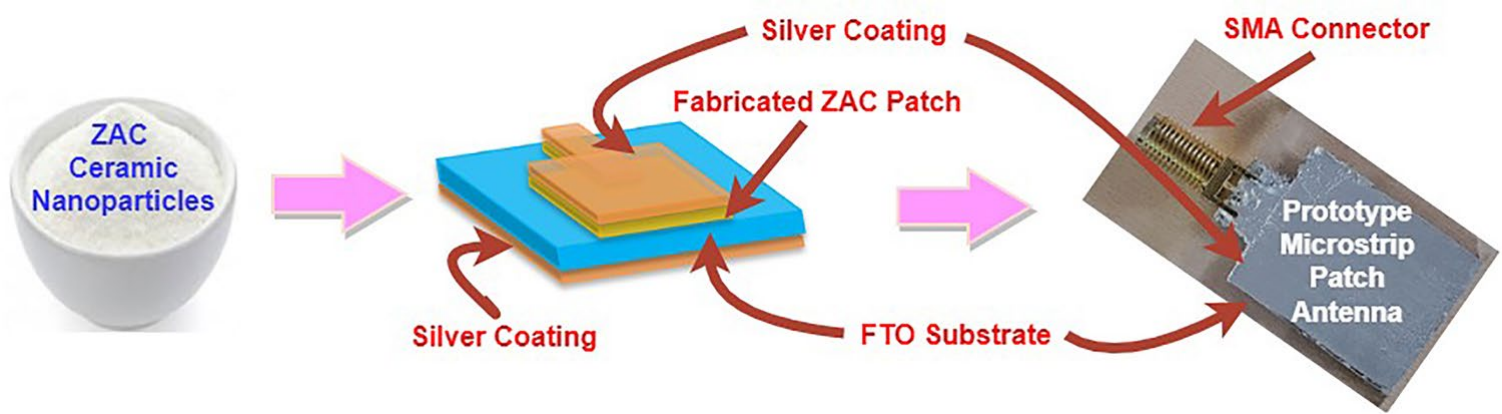
Process illustrating the preparation of ZAC ceramic nanoparticles.

Figure 8 provides an overview of the preparation process for the prototype antenna using ZAC ceramic nanoparticles. Initially, ceramic nanoparticles paste was applied onto the FTO (Fluorine-doped Tin Oxide) plate using the doctor blade method. This paste was then thermally treated to ensure adhesiveness and solidification processes. A layer of silver was applied over the previously prepared ZAC ceramic patch and the bottom of the substrate. This step is crucial to create a closed electrical circuit. In the next step, an SMA connector was connected and bonded to the antenna to enhance electrical conductivity, which is essential for antenna performance. Finally, a prototype MPA underwent performance testing.

**Figure 9:**
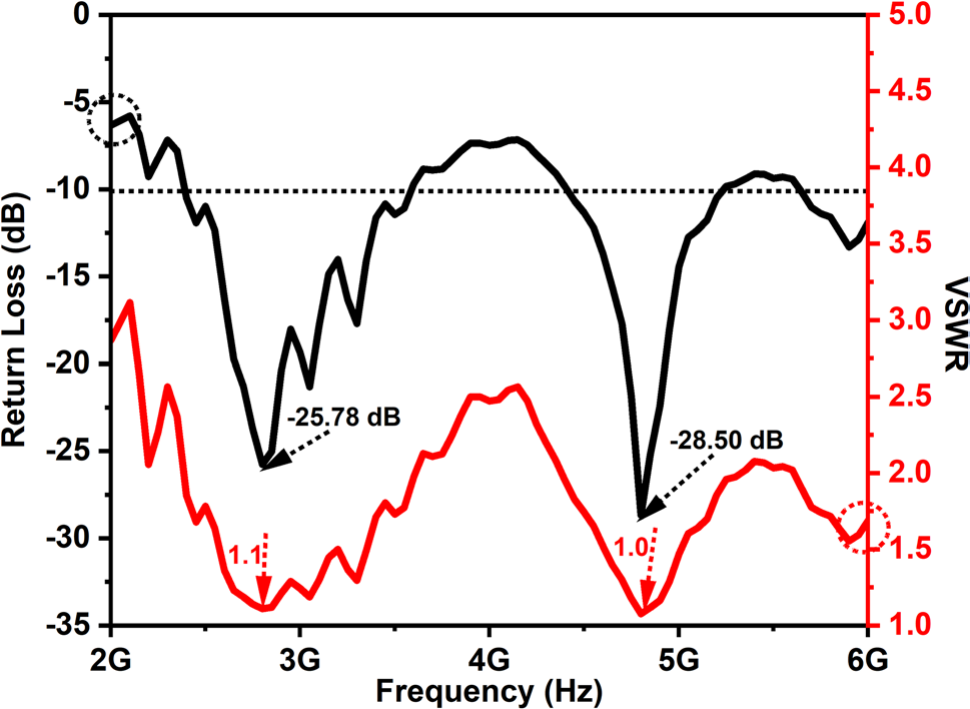
RL and VSWR of ZAC ceramic nanoparticles based microstrip patch antenna.

The return loss of an antenna quantifies the extent to which a signal is reflected back to the employed source, resulting from an impedance mismatch between the antenna and the transmission line. In other words, it tells us how much of the signal fed to the antenna is lost or reflected back instead of being transmitted. Figure 9 shows RL and VSWR characteristics of a prototype MPA prepared using ZAC ceramic nanoparticles in the frequency range from 2 to 6 GHz. This antenna exhibits two distinct resonant peaks, corresponding to the S-band and C-band frequencies. In specific, the return loss for the S-band was measured at − 25.78 dB, occurring at a resonant frequency of 2.8 GHz with a bandwidth of 1.2 GHz. For the C-band, the return loss was − 28.5 dB, resonating at 4.8 GHz with a bandwidth of 800 MHz.

These results demonstrate that the prepared ZAC material is well-suited for the fabrication of a dual-band suitable for both S- and C-band applications. A comparative study of dielectric properties of ceramics and microstrip patch antenna is shown in Table 3. Our antenna showed better performance as compared to others, which suggests the suitability of the prepared ZAC nanoparticles.

**Table 3:** Comparison of dielectric properties of dielectric ceramics and microstrip patch antennas.

Materials	Dielectric permittivity (ε_r_)	Dielectric loss (tanδ)	Return loss (dB)	Frequency (GHz)	VSWR	Band-width (MHz)	References
Na_0.5_Bi_0.5_MoO_4-_Li_2_MoO_4_	20.7	0.005	–	–	–	–	^39^
Bi_4_Ti_3_O_12_)_X-_(CaCu_3_Ti_4_O_12_)_1–X_	557	–	− 10.58	1.55	–	150	^40^
Mg_x_Cd_1−x_ Fe_2_O_4_	4.96	0.081	− 23.08	10.5	1.08	1500	^41^
CaTiO_3_-K_2_MoO_4_	8.5	0.0008	− 14.2	2.5	–	300	^42^
ZnAl_2_O_4_Ca	13	0.024	− 25.78 & − 28.5	2.8 & 4.8	1.1 & 1.01	1200 & 800	Present Work

VSWR is a measure of how effectively an antenna transfers power to the load. It is calculated using the equation $S=((|1+\rho |))/((|1-\rho |))$, where S is the standing wave ratio and ρ is the reflection coefficient. A low VSWR value signifies efficient power transfer from the antenna to the load, while a high VSWR value indicates poor power transfer and increased signal reflections. Ideally, a VSWR value close to 1 indicates a nearly perfect match between the antenna and the transmission line, resulting in minimal signal reflection. Figure 9 depicts the VSWR curve of the MPA plotted by varying frequency from 2–6 GHz. The VSWR values obtained were 1.1 for the S-band and 1.0 for the C-band resonant frequencies. These VSWR values suggest that the MPA is operating efficiently at both the S-band and C-band frequencies, indicating a well-matched antenna system with minimal power reflection.

MPAs possess numerous advantages, making them an excellent choice for designing and fabricating dual-band antennas. WLAN technology seamlessly integrates wireless communication into computer networks, utilizing the 2.4 GHz and 5 GHz spectrum within the Industrial Scientific Medical (ISM) band. The ability to use these frequency bands without requiring authorization has spurred the widespread adoption and application of WLAN, particularly due to advancements in wireless communication technology. It serves as a prevalent communication band in devices such as cell phones, smart televisions, computers, and laptops. This technology's user-friendly nature and its compatibility with printed MPA designs make it even more appealing^43^. Moreover, a dual-band MPA can be tailored to operate across two distinct frequency bands. This allows both communication systems to utilize the same antenna, leading to space savings and cost reduction. Nevertheless, fabricating a dual-band MPA that meets the requirements of both frequency bands poses a significant challenge, as the antenna must perform effectively across both frequency ranges^2^. In our case, the resonant frequencies closely coincide with those of WLAN antennas. This emphasizes the potentiality for further optimization of the antenna fabrication to meet the frequencies employed in WLAN applications.

## Conclusions

In this study, Ca-doped ZnAl_2_O_4_ dielectric ceramic nanoparticles were synthesized and analyzed. Materials characterization revealed the polycrystalline characteristics of the nanoparticles with a crystallite size of 8.1 nm, the presence of functional groups associated with the prepared ceramic compound and the spherical grains of ZAC with a mean diameter of 12.32 nm. Further, investigation of dielectric properties showed a decrease in dielectric permittivity and tangent loss along with enhanced conductivity with the rise in frequency. Finally, the fabricated prototype microstrip patch antenna demonstrated dual-band performance, with return losses of − 25.78 dB at 2.8 GHz (S-band) and − 28.5 dB at 4.8 GHz (C-band). VSWR values were measured to be 1.1 and 1.0 for the resonant frequencies of 2.8 GHz and 4.8 GHz, respectively. Our findings suggest the potential of Ca-doped ZnAl_2_O_4_ ceramic nanoparticles as alternate substrate material for fabricating dual-band microstrip patch antenna in WLAN applications.

## Data Availability

All data generated or analyzed during this study are presented in this manuscript and can be obtained upon reasonable request from the corresponding author.
