# Author Correction: Sol–gel derived ceramic nanoparticles as an alternative material for microstrip patch antenna in WLAN applications

**DOI:** 10.1038/s41598-024-66239-y

**Published:** 2024-07-11

**Authors:** Sekhar Didde, R. S. Dubey

**Affiliations:** 1 Department of Electronics & Communication Engineering, Swarnandhra College of Engineering and Technology, Seetharamapuram, Narsapur, A.P. India; 2 SR University, Ananthasagar, Hasanparthy, Hanumakonda District, Warangal, T.S. 506372 India

Correction to: *Scientific Reports* 10.1038/s41598-024-63454-5, published online 13 June 2024

The original version of this Article contained an error in Affiliation 1, which was incorrectly given as ‘Department of ECE, Koneru Lakshmaiah Education Foundation, Greenfields, Vaddeswaram, Guntur, A.P., India.’ The correct Affiliation is listed below:

Department of Electronics & Communication Engineering, Swarnandhra College of Engineering and Technology, Seetharamapuram, Narsapur, (A.P.), India.

Additionally, the Reference, which is listed below as Reference 26, and the reprint permission for Figure 1, were omitted.

26. Didde, S., Dubey, R.S., Panda, S.K. *et al.* Experimental study of doped zinc aluminate nanoparticles by bottom-up approach for microstrip patch antenna applications. *J Mater Sci* **57**, 21069–21079 (2022). 10.1007/s10853-022-07929-8

The caption of Figure 1 now reads:

“The process of preparation of ZAC ceramic nanoparticles. Reprinted with permission of Springer Nature © 2022^26^.”

As a result of the changes, the References have been renumbered.

The original Article has been corrected.

